# Developing an internal threshold of toxicological concern (iTTC)

**DOI:** 10.1038/s41370-022-00494-x

**Published:** 2022-11-08

**Authors:** Jon A. Arnot, Liisa Toose, James M. Armitage, Alessandro Sangion, Alexandra Looky, Trevor N. Brown, Li Li, Richard A. Becker

**Affiliations:** 1 ARC Arnot Research and Consulting Inc., Toronto, ON Canada; 2 Department of Physical and Environmental Sciences, University of Toronto Scarborough, Toronto, ON Canada; 3 Department of Pharmacology and Toxicology, University of Toronto, Toronto, ON Canada; 4 School of Public Health, University of Nevada Reno, Reno, NV USA; 5 American Chemistry Council, Washington, DC USA

**Keywords:** Exposure modeling, Dermal exposure, Dietary exposure, Inhalation exposure, New approach methodologies (NAMs), PBPK modeling

## Abstract

**Background:**

Threshold of Toxicological Concern (TTC) approaches are used for chemical safety assessment and risk-based priority setting for data poor chemicals. TTCs are derived from in vivo No Observed Effect Level (NOEL) datasets involving an external administered dose from a single exposure route, e.g., oral intake rate. Thus, a route-specific TTC can only be compared to a route-specific exposure estimate and such TTCs cannot be used for other exposure scenarios such as aggregate exposures.

**Objective:**

Develop and apply a method for deriving internal TTCs (iTTCs) that can be used in chemical assessments for multiple route-specific exposures (e.g., oral, inhalation or dermal) or aggregate exposures.

**Methods:**

Chemical-specific toxicokinetics (TK) data and models are applied to calculate internal concentrations (whole-body and blood) from the reported administered oral dose NOELs used to derive the Munro TTCs. The new iTTCs are calculated from the 5th percentile of cumulative distributions of internal NOELs and the commonly applied uncertainty factor of 100 to extrapolate animal testing data for applications in human health assessment.

**Results:**

The new iTTCs for whole-body and blood are 0.5 nmol/kg and 0.1 nmol/L, respectively. Because the iTTCs are expressed on a molar basis they are readily converted to chemical mass iTTCs using the molar mass of the chemical of interest. For example, the median molar mass in the dataset is 220 g/mol corresponding to an iTTC of 22 ng/L-blood (22 pg/mL-blood). The iTTCs are considered broadly applicable for many organic chemicals except those that are genotoxic or acetylcholinesterase inhibitors. The new iTTCs can be compared with measured or estimated whole-body or blood exposure concentrations for chemical safety screening and priority-setting.

**Significance:**

Existing Threshold of Toxicological Concern (TTC) approaches are limited in their applications for route-specific exposure scenarios only and are not suitable for chemical risk and safety assessments under conditions of aggregate exposure. New internal Threshold of Toxicological Concern (iTTC) values are developed to address data gaps in chemical safety estimation for multi-route and aggregate exposures.

## Introduction

Hazard and exposure data required for the human health assessment of thousands of chemicals are limited. Risk-based chemical priority setting methods are being developed and applied to identify those chemicals that pose the greatest health concern to focus resources and assessment efforts [1–5]. New Approach Methods (NAMs) are being developed and applied to reduce animal testing and address hazard and exposure data gaps, including the US Environmental Protection Agency’s ToxCast [6] and ExpoCast [7–9] programs and the Exposure And Safety Estimation (EAS-E) Suite platform (www.eas-e-suite.com). The Threshold of Toxicological Concern (TTC) is a well-established approach [10–14] used to address hazard data gaps by regulatory agencies such as Health Canada [15], the European Food Safety Agency (EFSA) [16], and the US Food and Drug Administration [17]. In the absence of chemical-specific toxicity data, the TTC approach provides an estimate for a level of exposure in which no appreciable human health risk is expected [18]. A review by EFSA and the World Health Organization (WHO) [19] concluded that the TTC is a fit-for-purpose approach with broad applicability for risk estimation. Subsequently, Patlewicz et al. [20] selected a daily oral exposure-based TTC and high throughput exposure predictions from ExpoCast for approximately 8000 chemicals to demonstrate a risk-based priority setting method.

Humans can be exposed simultaneously from multiple exposure pathways, i.e., aggregate exposure, and this limits the application of route-specific TTCs such as a daily oral exposure-based TTC. For example, Health Canada discusses the challenge in applying an oral TTC value for screening risk evaluations to chemicals for which exposure occurs by other routes, such as dermal or inhalation [15]. Current TTCs derived from in vivo experimental data are exposure-route specific. For example, oral (ingestion) based TTCs were first developed 15–20 years ago [11, 12, 14]. Following the general approach for ingestion based TTCs, inhalation TTCs, e.g., [21], and dermal TTCs, e.g., [22], have also been developed. Partosch et al. [23] proposed a route-to-route extrapolation method by revising the TTC to account for oral bioavailability; however, this proposed method does not adequately address the situation because it ignores other toxicokinetic (TK) processes [24, 25]. The concept of an internal TTC (iTTC) has been proposed, e.g., [26–28] to address current limitations of the TTC so the approach can be applied more broadly including comparisons with aggregate exposure estimates. Essentially iTTCs can be determined from the same data used to derive external TTCs by applying TK data and models to convert external exposure doses to internal exposure doses, e.g., concentrations in blood or plasma [27, 28]. However, as highlighted in proposed iTTC workflows, e.g., [27–29] one of the principal limitations in applying TK models in this manner is the paucity of chemical-specific TK data, e.g., biotransformation rates, for all chemicals in the TTC databases. The ExpoCast program and other research have advanced TK modeling for data poor chemicals using in silico and in vitro NAMs for high throughput toxicokinetic (HTTK) and exposure models, e.g., [8, 9, 30–33]. Advancements include the development and validation of quantitative structure-activity relationships (QSARs) for predicting total (terminal) elimination half-lives and whole-body biotransformation half-lives in humans (HL_T_ and HL_B_, respectively) [34, 35] for parameterizing TK models for applications in regulatory decision-making [36].

This study applies available TK data and models to derive in vivo iTTCs for whole-body and blood using No Observed Effect Levels (NOELs) reported for oral exposures. This approach is demonstrated as a case study with the NOEL dataset used to derive the Munro oral exposure TTCs [11]. A tiered approach for applying TK data to estimate steady-state internal concentrations preferentially uses measured in vivo TK data when available and model estimates for TK parameters when measured in vivo data are lacking. A one-compartment physiologically-based toxicokinetic (1-CoPBTK) model for mammals parameterized with available in vitro, in vivo and in silico data is used to estimate chemical-specific TK parameters in the absence of measured in vivo TK data. Cumulative distributions of the new internal exposure concentrations corresponding to the Munro NOELs are used to select the 5th percentile values from which new iTTCs for whole-body and blood are calculated with commonly applied uncertainty factors. Recommendations for improving the approach and to expand and better define the chemical applicability domain of the new iTTCs are provided.

## Methods

### General toxicokinetic model

**Fig. 1:**
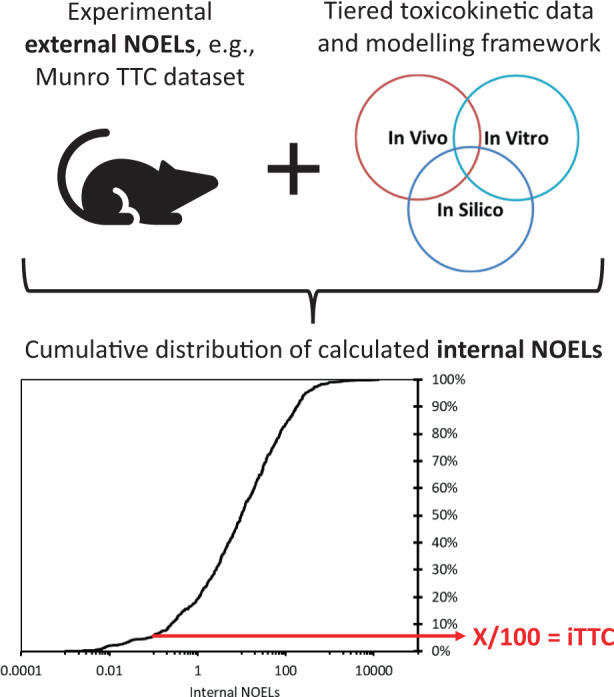
Conceptual overview of the approaches used in this study to derive iTTCs. External dose NOELs were converted using a general toxicokinetic model to generate the cumulative distribution of internal NOELs, and then lower 5th percentile internal NOEL value was divided by a 100-fold adjustment factor to calculate the iTTC.

Figure 1 provides a conceptual overview of the general methods developed and applied to calculate internal concentrations from exposure doses and to derive new whole-body and blood iTTCs. The fundamental relationship between external exposures (used for TTCs) and internal exposures (used for iTTCs) is determined by chemical- and organism-specific toxicokinetics. An internal whole-body exposure (dose) can be calculated from an external oral ingestion rate (dose) as:

1 $${{{{{{{\mathrm{C}}}}}}}}_{{{{{{{{\mathrm{WB}}}}}}}}} = {{{{{{{\mathrm{OD}}}}}}}}\,{{{{{{{\mathrm{x}}}}}}}}\,{{{{{{{\mathrm{AE}}}}}}}}/{{{{{{{\mathrm{k}}}}}}}}_{{{{{{{\mathrm{T}}}}}}}} = {{{{{{{\mathrm{NOEL}}}}}}}}\,{{{{{{{\mathrm{x}}}}}}}}\,{{{{{{{\mathrm{AE}}}}}}}}/{{{{{{{\mathrm{k}}}}}}}}_{{{{{{{\mathrm{T}}}}}}}}$$

where C_WB_ is the steady-state whole-body concentration (mg/kg-body), OD is the oral dose (mg/kg-body/d), AE is the chemical absorption efficiency (unitless) from the gastrointestinal tract (GIT), and k_T_ is the first-order whole-body total (terminal) elimination rate constant (1/d). In the current application of the models, OD is replaced with reported NOEL (mg/kg-body/d). The AE parameter is different from oral bioavailability (F; often expressed as a percentage) because F includes first pass effects in the liver and AE quantifies chemical absorption from the GIT lumen into blood (portal vein) [37]. The k_T_ parameter is the sum of individual chemical elimination process rate constants as:

2 $${{{{{{{\mathrm{k}}}}}}}}_{{{{{{{\mathrm{T}}}}}}}} = {{{{{{{\mathrm{k}}}}}}}}_{{{{{{{\mathrm{R}}}}}}}} + {{{{{{{\mathrm{k}}}}}}}}_{{{{{{{\mathrm{F}}}}}}}} + {{{{{{{\mathrm{k}}}}}}}}_{{{{{{{\mathrm{U}}}}}}}} + {{{{{{{\mathrm{k}}}}}}}}_{{{{{{{\mathrm{B}}}}}}}} + {{{{{{{\mathrm{k}}}}}}}}_{{{{{{{\mathrm{G}}}}}}}}$$

where k_R_, k_F_, k_U_ and k_B_ are rate constants for respiratory elimination, fecal egestion, urinary excretion (renal clearance), and biotransformation (metabolism), respectively. These rate constants constitute the primary chemical elimination processes in an organism. For persistent chemicals that are very slowly eliminated and very slowly biotransformed, organism growth can influence the concentration, therefore, k_G_ (rate constant to account for growth) can be included as a “pseudo” elimination rate process in the steady-state calculation. k_T_ can be measured in vivo or calculated using PBTK models. First order rate constants operating at an organism level can be converted to whole-body half-lives and vice versa, e.g., HL_T_ = ln2/k_T_ and HL_B_ = ln2/k_B_. Steady-state blood concentrations (C_B_; mg/L-blood) can be calculated from C_WB_ using the steady-state volume of distribution referenced to blood (V_D_; L-blood/kg-body) as:

3 $${{{{{{{\mathrm{C}}}}}}}}_{{{{{{{\mathrm{B}}}}}}}} = {{{{{{{\mathrm{C}}}}}}}}_{{{{{{{{\mathrm{WB}}}}}}}}}/{{{{{{{\mathrm{V}}}}}}}}_{{{{{{{\mathrm{D}}}}}}}}$$

### Munro TTC dataset

The NOELs from the original Munro database derived from administered oral doses were used in Eq. 1, i.e., OD = NOEL, to calculate internal dose C_WB_ and C_B_. The original Munro oral TTC values were derived from 613 organic chemicals associated with 2941 NOELs from non-cancer endpoints in toxicity studies conducted in rats (*n* = 489), mice (*n* = 90), hamsters (*n* = 31), and rabbits (*n* = 3). As detailed in Munro et al. [11] the 613 chemicals were assigned to three Cramer classes based on the Cramer classification scheme [38] (i.e., Class I – low; *n* = 137, Class II – medium; *n* = 28, Class III – high level of concern; *n* = 448). A TTC for each Cramer class used by Munro et al. [11] was determined using the 5th percentile NOEL and dividing by an uncertainty factor of 100 to obtain intake rate based TTCs of 30, 9.0 and 1.5 µg/kg-bw/d for Cramer class I, II and III, respectively for a 60 kg adult. The “original” Munro dataset [11] as reported by Bassan et al. [39] which includes Simplified Molecular Input Line Entry System (SMILES) notations [40] was used. The Munro et al. NOEL toxicity data summarized by Bassan et al. [39] were not critically evaluated; however, a cursory analysis of the SMILES associated with the database was conducted. The exposure dose was determined as chemical quantity (e.g., mmol-chemical/kg/d), not mass (e.g., mg-chemical/kg/d) because it is the quantity of chemical, not its mass, at a site of toxic action that may elicit a biological response. Further comments on the Munro data are in the Supplementary Information (SI). Chemicals identified as genotoxic or acetylcholinesterase inhibitors as used in other TTCs were not included here.

### Model parameterization

**Fig. 2:**
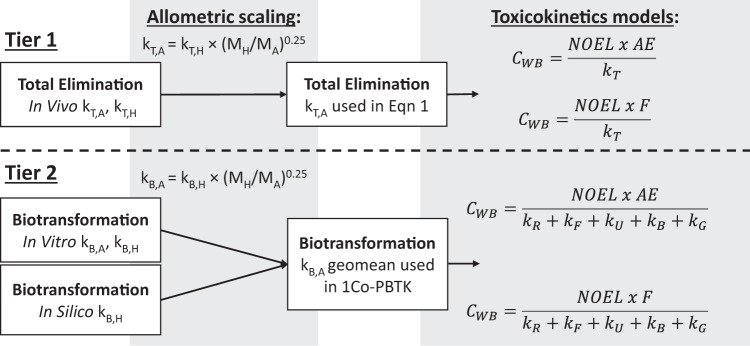
Tiered approach and workflow for parameterizing toxicokinetics models with whole-body total (terminal) elimination rate constant (k_T_) and whole-body biotransformation rate constant (k_B_) data to calculate internal doses from the reported NOELs in the Munro TTC database [11, 39] as derived from external oral doses. Blood concentrations (C_B_ or C_blood_) are then calculated from whole body concentrations (C_WB_) as shown in Eq. (3). Absorption efficiency (AE) is used in the parameterization of the default and alternative #1 models while oral bioavailability (F) is used in the alternative model #2 calculations.

Figure 2 shows tiers of TK data used to parameterize k_T_ in Eq. 1 to estimate internal doses (C_WB_ and C_B_) from the NOELs reported in the Munro TTC dataset [11, 39]. In Tier 1 measured in vivo k_T_ data are used to parameterize Eq. 1. For chemicals without in vivo k_T_ data, Tier 2 uses the 1-CoPBTK model parameterized with in vitro and in silico (QSAR) estimates for k_B_ to calculate k_T_ that is then used in Eq. 1. The 1-CoPBTK model is generic in the sense that it can be readily parameterized for (i) many mammalian species requiring only minimal organism specific parameters to predict physiological processes, and (ii) many organic chemicals requiring only minimal chemical-specific parameters, e.g., k_B_ and distribution ratios. The 1Co-PBTK model is part of a general modeling framework that has been applied and evaluated for various mammals over the past 20 years, e.g., [33, 34, 41–44]. Details of the 1-CoPBTK model are presented in the SI. The 1Co-PBTK models are coded in the EAS-E Suite HTTK module (www.eas-e-suite.com) for HTTK applications for an adult male human and rat.

A literature search for measured in vivo mammalian TK data for the chemicals in the Munro database [11] was conducted. Rat in vivo k_T_ data were selected preferentially for toxicity studies for rats, and mouse in vivo k_T_ were selected preferentially for toxicity studies using mice; however, if there were no rat data for a chemical but mouse data were available then the in vivo mouse data were used and vice versa using allometric scaling. In vivo human k_T,H_ values were scaled to test animal specific k_T,A_ values using the following allometric relationship:

4 $${{{{{{{\mathrm{k}}}}}}}}_{{{{{{{{\mathrm{T,A}}}}}}}}} = {{{{{{{\mathrm{k}}}}}}}}_{{{{{{{{\mathrm{T,H}}}}}}}}} \times \left( {{{{{{{{\mathrm{M}}}}}}}}_{{{{{{{\mathrm{H}}}}}}}}/{{{{{{{\mathrm{M}}}}}}}}_{{{{{{{\mathrm{A}}}}}}}}} \right)^{0.25}$$

where M_H_ is the human body mass (70 kg) assumed for the empirical k_T,H_ data and M_A_ is the experimental animal body mass (assumed 0.025 kg for mouse, 0.25 kg for rat and hamster, and 5 kg for rabbit studies).

In absence of any empirical in vivo k_T_ data, the Tier 2 methods for parameterizing Eq. (1) were applied. In Tier 2 test animal specific k_T,A_ values were calculated using the 1-CoPBTK model with k_B,A_ estimates from (i) hepatocyte assays and common in vitro-in vivo extrapolation (IVIVE) models and (ii) QSARs for predicting HL_B,H_ (in humans). Details of the IVIVE calculations for scaling in vitro biotransformation rates to k_B_ are provided in the SI. The five HL_B,H_ QSARs [34, 35] used here were developed from in vivo human data as described by Arnot and colleagues [34]. Predicted HL_B,H_ values were converted to k_B,H_ as k_B,H_ = ln2/HL_B,H_ and k_B,H_ values were then scaled to toxicity test animal body mass values (k_B,A_) using allometric scaling Eq. 4 replacing k_T_ with k_B_. The QSARs have been developed and validated following OECD guidance for QSAR applications in regulatory decision-making [36]. The QSAR predictions include Applicability Domain (AD) information as detailed in the SI. When in vitro biotransformation rates were available from rodents or humans the IVIVE estimates of k_B_ were combined with “in” domain HL_B_-QSAR predictions to calculate a geometric mean for k_B_ that was then used to parameterize the test animal 1-CoPBTK models. If in vitro biotransformation rates were not available, the geometric mean of the “in” domain QSAR predictions for k_B_ were calculated to parameterize the 1-CoPBTK models.

#### Alternative TK model assumptions

Equation (1) requires an estimate for oral AE. An AE model that is primarily a function of the octanol-water partition coefficient (K_OW_) and chemical residence time in the GIT [45] was used in the default calculations (see SI for details). One principal limitation when applying the general TK model described in Eq. 1 is the absence of a first-pass effect in the liver. The parameter AE in Eq. (1) considers absorption from the GIT into the portal vein and does not explicitly account for the potential reduction in systemic blood concentrations (and whole-body concentrations) that may occur if there is significant biotransformation in GIT tissues or the liver. While AE is conservative for exposure assessment it may be inappropriate when estimating internal hazard values from external doses. Empirical estimates of bioavailability (F) from the literature from studies with mammals (humans, rats) were obtained to address the potential error of ignoring first-pass effects in the default model parameterization. In the absence of empirical estimates of F, predictions from the ACD Labs model (Release 2019.2.1, Build 3285. 16 Jan 2020) were obtained. The general model was then parameterized with F instead of AE for a second set of calculations and this is referred to as alternative model #1. For the TK model calculations, a minimum value of 1% was set for AE and F when estimates were < 1%.

The HTTK model used to estimate k_T_ for the test animals includes two options for predicting renal clearance. The first option used in the default model assumes urine excretion is a function of equilibrium partitioning between the body and urine as is commonly used in 1-CoPBTK models used for environmental exposure estimation [33, 34, 41, 44]. A second option assumes the glomerular filtration rate methods that are more commonly used in IVIVE TK models [31, 32]. The glomerular filtration prediction method results in faster rates of renal clearance compared to the partitioning model for more water-soluble chemicals, i.e., log K_OW_ < 3. When urinary excretion of chemicals is faster, k_T_ becomes faster resulting in lower predicted internal concentrations. To address some of the uncertainty in renal elimination rates an additional set of simulations using the glomerular filtration calculation method was combined with the assumption of using F instead of AE and this approach is referred to as alternative model #2.

Hepatic blood flow limitations can occur for chemicals that are rapidly biotransformed in the liver and biotransformed slowly, or not at all, in other compartments of the body. Under these conditions the net effect of error is that k_T_ values calculated from a 1-CoPBTK model can be faster than k_T_ values calculated from a multicompartment PBTK model parameterized with hepatic clearance estimates only [44]. Many chemicals are biotransformed in other compartments of the body and for such chemicals the potential hepatic blood-flow limitations could be irrelevant. It is not currently possible to explicitly address the potential errors that may exist in the 1-CoPBTK model k_T_ calculations for these conditions because the uncertainty in potential extra-hepatic clearance is not known for most chemicals in the Munro database. Fortunately, in the absence of empirical k_T_ data, the approach here uses whole-body estimates of biotransformation (k_B_) that account for hepatic as well as extra-hepatic biotransformation [34].

#### Physical-chemical properties

Physical-chemical properties required to parameterize the generic 1-CoPBTK models include molar mass, pKa (for ionizable organics), K_OW_ and octanol-air partition coefficients (K_OA_). Chemical data are summarized in the SI.

## Results

### Model input parameters

#### Munro dataset

The NOELs in the Munro TTC dataset span about 6 orders of magnitude ranging from 0.005 (Haloxyfop-methyl) to 7,203 (Calcium cyclamate) mg/kg/d with a median value of 30 mg/kg/d. When the NOELs were converted to a molar basis for the discrete organic moiety, the external NOELs span about 7 orders of magnitude ranging from 1.3 × 10^−5^ (dieldrin) to 114 mmol/kg/d (ethanol) with a median of 0.14 mmol/kg/d. When expressed as external molar concentrations some of the chemicals with the lowest NOEL doses (~ 10^−5 ^mmol/kg/d) are dieldrin, haloxyfop-methyl, abamectin B1, zeranol. Chemicals with some of the highest external NOEL doses (~ 10–100 mmol/kg/d) include alcohols, e.g., ethanol, methanol, glycerol, propylene glycol.

The Munro TTC dataset comprises a diverse range of chemical classes and properties. The molar mass of the administered chemicals (including salt formulations) ranges from 30 to 1,135 g/mol with a median of 223. The molar mass of the discrete organic molecules in the dataset ranges from 23 to 1,135 g/mol with a median of 220. For the discrete organic chemical structures, the measured log K_OW_ values (*n* = 402/613) range from −5.4 to 10.0 and the geometric means of the predicted log K_OW_ values range from −6.0 (β-cyclodextrin) to 16.2 (stearyl tartrate) for the remaining chemicals. Due to a general lack of measured K_OA_ data, the K_OA_ values required to parameterize the 1-CoPBTK model were predicted from QSARs and polyparameter linear free energy relationship (ppLFERs). The geometric means of the predicted log K_OA_ values range from 1.2 to 25. One hundred ninety-six of the chemicals were classified as mono-protic acids with pKas ranging from −3.6 to 11.3, with a median of 4.6. Fifty-eight of the chemicals were classified as mono-protic bases with pKas ranging from 4.0 to 13.6, with a median of 7.6.

#### Summary of in vitro, in vivo and in silico TK data

In vivo rat and mouse HL_T_ data were found for 38 and 4 chemicals in the Munro database, respectively. In vivo HL_T_ data from human studies were found for 63 chemicals. Combined there were empirical in vivo HL_T_ estimates for 91 of the 613 chemicals. In vitro rat and human hepatocyte biotransformation rates were obtained for 18 and 102 chemicals, respectively. The in vitro biotransformation rates were critically evaluated for data quality using methods and criteria described elsewhere [46]. Combined there are in vitro measurements for 111 of the chemicals for which there were no in vivo HL_T_ measurements. Whole-body biotransformation half-lives (HL_B_) predicted by QSARs [34, 35] that were within their defined applicability domains were used for 522 of the 613 Munro TTC database chemicals.

The measured in vivo HL_T_ data (at 0.25 kg-bw) range from 0.02 to 4700 hours with a median of 1.6 h. The in vitro and in silico HL_T_ data (at 0.25 kg-bw) range from 0.02 to 8,100 hours with a median of 1.1 h. Combining the in vivo with the in vitro and in silico estimates, the HL_T_ values used to estimate internal exposures range from 0.02 to 4700 hours (spanning about 6 orders of magnitude) with a median of 1.1 h. The distribution of estimated HL_T_ is very similar to the distribution of empirical HL_T_. Chemicals with the shortest HL_T_ (ca. a few minutes) include formaldehyde, formic acid, and vinyl chloride. Chemicals with the longest HL_T_ (ca. 60 to 200 d) include Persistent Organic Pollutants (POPs) listed under the Stockholm Convention like PCBs, mirex, polybrominated diphenyl ethers (PBDEs), p,p-DDT, lindane, dieldrin, and hexachlorobenzene.

Empirical estimates of oral bioavailability (F) from experimental studies with mammals were found for 53 chemicals (see TK databases in EAS-E Suite; www.eas-e-suite.com). The empirical estimates for F range from 5 to 100% (median = 74%) and are in reasonable agreement with the default AE model calculations and the ACD Labs F calculations. The net result of applying the alternative model #1 is a lower value in the numerator (Eq. 1) and hence a lower predicted internal concentration for chemicals when F is lower than AE. Most potential errors in estimating AE and F are considered relatively minor in the current context because if for example the true value is 50% instead of 100% then the predicted steady-state internal exposure concentrations are overestimated by only a factor of 2.

### Model calculations

#### Default model

The calculated whole-body concentrations for the database range from 9.9×10^−4^ to 1.3×10^4^ µmol/kg (spanning over 7 orders of magnitude) with a median of 9.7 µmol/kg. The calculated molar blood concentrations for the database range from 3.2×10^−4^ to 2.1×10^3^ µmol/L-blood (spanning almost 7 orders of magnitude) with a median of 3.9 µmol/L-blood. When expressed as internal molar concentrations (whole-body or blood), many of the more potent chemicals in the dataset are known bioactive chemicals and biocides, e.g., glufosinate, abamectin B1, zeranol, haloxyfop-methyl, tetrakis(hydroxymethyl)phosphonium, and paraquat. Some examples of chemicals with the highest internal molar doses (indicating relative lower potency) are those often included in food, e.g., quercetin, various simple alcohols, some dyes (food additives), and relatively unreactive chemicals like DDT, PBDEs, and PCBs.

#### External and internal chemical concentration comparisons

**Fig. 3:**
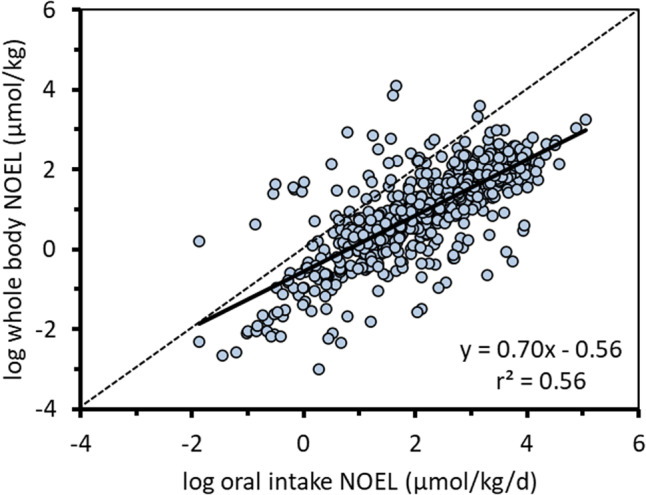
External and internal doses corresponding to NOELs in the Munro TTC database [11, 39]. The dashed diagonal line represents the 1:1 line.

Figure 3 compares administered external doses (µmol/kg/d) and internal whole-body concentration doses (µmol/kg) corresponding to the reported NOELs for the parent (discrete organic moiety) chemicals (*n* = 613). The internal concentrations include explicit consideration for chemical-specific toxicokinetics (i.e., absorption, elimination, and biotransformation of parent chemical) and thus the relative potencies are more directly comparable between chemicals in the internal dose dataset. While there is a positive correlation between the two datasets, the external dose only explains about 56% of the chemical potency, the rest can be explained by toxicokinetics. Many chemicals that appear to be relatively “more toxic” because of lower oral intake NOELs are actually relatively “less toxic” than other chemicals with respect to internal dose. In some cases, the apparent toxicity and relative potency as indicated by the two approaches differ by several orders of magnitude reflecting the influence of HL_T_, and to some degree AE, on internal exposure.

#### Alternative models

The model that considers some uncertainty in possible first-pass effects (alternative model #1) calculated whole-body molar concentrations ranging from 7.4×10^−4^ to 3.9×10^3^ µmol/kg with a median of 6.2 µmol/kg. The molar blood concentrations range from 3.1×10^−4^ to 2.0×10^3^ µmol/L-blood with a median of 2.3 µmol/L-blood. As expected, the internal concentrations for some chemicals are lower and the median concentrations are slightly less than twofold lower compared to the default model. The model that includes the potential for first-pass effects and higher rates of urinary excretion for lower K_OW_ chemicals (alternative model #2) calculated whole-body molar concentrations ranging from 6.3×10^−4^ to 3.8×10^3^ µmol/kg with a median of 5.5 µmol/kg. The calculated molar blood concentrations from 3.1×10^−4^ to 2.0×10^3^ µmol/L-blood with a median of 2.0 µmol/L-blood. Here again for some chemicals the internal concentrations decrease but the distributions remain largely similar compared to the default and alternative #1 model calculations.

### Deriving new TTCs and iTTCs

In this case study the database was not split into the three Cramer Classes associated with Munro TTCs so that the new iTTCs could be applied generally to organic chemicals that are not identified as being genotoxic or acetylcholinesterase inhibitors, i.e., certain carbamates and organophosphates, thus minimizing the need for decision-trees for an initial screening. The fifth percentile of the cumulative distribution of all NOELs in the Munro database expressed on a chemical mass oral intake rate basis is 280 µg/kg/d (not shown). Applying an assumed uncertainty factor of 100 consistent with previous methods for deriving TTCs from this dataset, the TTC is 2.8 µg/kg/d. This TTC for the entire database is included for context when comparing against other TTCs and iTTCs calculated in this study, and not as another TTC for consideration for regulatory applications. For example, the TTC based on the total Munro dataset is more conservative than the Cramer Class I and II TTCs and within a factor of 2 of the Cramer Class III TTC (1.5 µg/kg/d). The fifth percentile of the cumulative distribution of all NOELs expressed on a molar basis for the discrete organic molecules is 0.83 µmol/kg/d (not shown). Applying an uncertainty factor of 100 to this value results in a TTC of 0.0083 µmol/kg/d. Recall the range of molar mass for administered chemicals in the dataset is large, but the median is 220 g/mol approximating the difference between the two TTCs. The actual factor difference between the mg and mmol oral intake TTCs is 337 which is a result of treating the discrete organic molecules separately from the administered salt formulations (see SI for details).

**Fig. 4:**
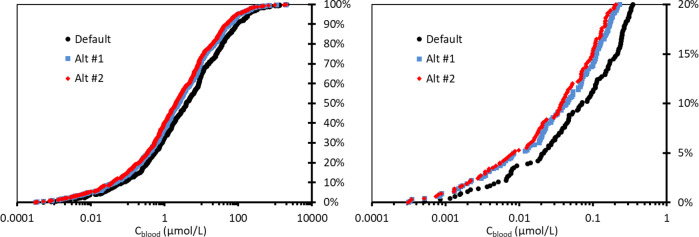
Cumulative distribution of modeled blood concentrations corresponding to NOELs reported in the Munro TTC database [11, 39] using the default and alternative assumptions (Alt #1, Alt #2, see “Methods” section). The figure on the right provides a better view of the lower 20% of the data in the cumulative distribution.

Table 1 summarizes the new iTTCs. Figure 4 shows the cumulative distributions of the blood concentrations for the discrete organic molecules (*n* = 613) calculated from the Munro TTC NOELs using the three different model assumptions. There is general consistency between the 3 distributions and the alternative models #1 and #2 show slightly lower blood concentrations largely reflecting the differences in assumptions relating to reduced uptake and enhanced renal clearance. The 5^th^ percentiles of steady-state blood concentrations are 22, 8.5 and 8.3 nmol/L for the default, alternative #1 and alternative #2 model calculations, respectively. Following the general operating procedures for applying safety (uncertainty) factors for calculating TTCs from NOELs an uncertainty factor of 100 was applied to account for interspecies differences (factor of 10) and human variability (factor of 10) [47–49]. The new blood iTTCs are 0.22, 0.085 and 0.083 nmol/L for the default, alternative #1 and alternative #2 model calculations, respectively. Given the consensus in the iTTCs from the three models and the underlying uncertainty in the hazard and TK data a blood iTTC of 0.1 nmol/L (or pmol/mL-blood) is selected for organic chemicals that are not considered genotoxic and are not acetylcholinesterase inhibitors. This iTTC is therefore rather broadly applicable for a range of organic chemicals. Furthermore, because the new iTTC is expressed on a molar basis it can be readily converted to an iTTC expressed on a chemical mass basis by multiplying 0.1 nmol/L by the molar mass of the chemical of interest, for example when comparing to human exposure blood concentrations expressed in units of ng/L or pg/mL. As a first approximation, using the median value for molar mass in the dataset (220 g/mol) corresponds to a blood iTTC of 22 ng/L-blood (or 22 pg/mL-blood).

**Table 1:** Summary of new iTTCs derived in this case study.

	Modeling approach			
	Default	Alternative #1	Alternative #2	Selected*
Blood iTTC (nmol/L)	0.22	0.085	0.083	0.10
Whole-body iTTC (nmol/kg)	0.74	0.29	0.23	0.50

*Given the general uncertainty in the hazard and TK data, a value approximating central tendency from the three estimation methods is selected and recommended for iTTCs for organic chemicals other than those indicating genotoxicity or acetylcholinesterase inhibition.

**Fig. 5:**
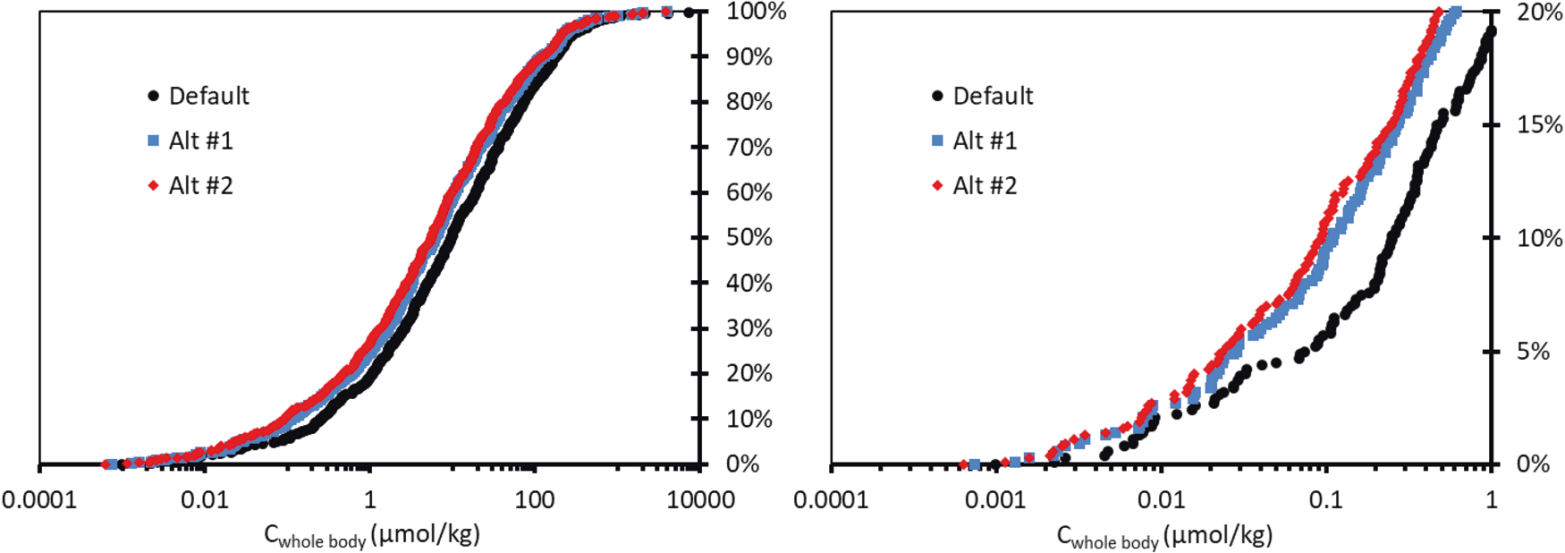
Cumulative distribution of modeled whole-body concentrations corresponding to NOELs reported in the Munro TTC database [11, 39] using the default and alternative assumptions (Alt #1, Alt #2, see “Methods” section). The figure on the right provides a better view of the lower 20% of the data in the cumulative distribution.

Figure 5 shows the cumulative distributions of the whole-body concentrations for the discrete organic molecules (*n* = 613) calculated from the NOELs using the three different model assumptions. As with blood, there is general consistency between the 3 distributions and the alternative models #1 and #2 show slightly lower concentrations. The 5th percentiles of steady-state whole-body concentrations are 0.074, 0.029 and 0.023 µmol/kg for the default, alternative #1 and alternative #2 model calculations, respectively. Applying an uncertainty factor of 100 results in new whole-body iTTCs of 0.74, 0.29 and 0.23 nmol/kg, respectively. Given the consensus in the iTTCs from the three models a whole-body iTTC of 0.5 nmol/kg is selected for organic chemicals that are not genotoxic and are not acetylcholinesterase inhibitors. This whole-body iTTC is therefore rather broadly applicable for a range of organic chemicals. As with the new blood iTTC, the whole-body iTTC can be expressed on a chemical mass basis by multiplying 0.5 nmol/kg by the molar mass (g/mol) of the chemical of interest. For example, using the median value for molar mass in the dataset (220 g/mol) corresponds to a whole-body iTTC of 110 ng/kg-bw. While the blood iTTC will likely be more applicable to human health studies, the whole-body iTTC may be helpful for applications to non-human receptors or when exposure estimates can only be obtained at a whole-body level.

#### Examining the steady-state assumption in the Munro TTC database

It has been recommended by experts at EFSA [49] that the TTC approach is not applicable “for chemicals that are known or predicted to bioaccumulate.” However, there was no practical or quantitative guidance provided for determining what constitutes a “bioaccumulative chemical” for exclusion. Leeman et al. [50] highlight that the Munro TTC database includes many POPs and “bioaccumulative chemicals” including PCBs (Arochlor 1254), PBDEs, chlordane, p,p-DDT, mirex and hexachlorobenzene [11]. Kroes et al. [14] had previously indicated that bioaccumulation is not accounted for in the TTC approach. We analyzed the experimental NOEL data to determine if the exposure durations in the sub-chronic and chronic experiments were sufficiently long for the organism to approach steady-state. The time to approach 95% of steady-state can be estimated as 4 × HL_T_. Using the in vivo, or default model estimates for HL_T_ when necessary, and the reported experimental exposure durations, all but three chemicals are expected to approach 95% of steady-state within the experimental test duration (SI Section S5). Thus, from a hazard threshold perspective, the Munro TTCs can be considered “valid” with respect to the steady-state assumption and the exclusion of the TTC approach for bioaccumulative chemicals seems unwarranted.

## Discussion

The Munro dataset comprises a diverse range of chemical classes, structures, physical-chemical properties, and biological half-lives. The new in vivo iTTCs for whole-body and blood concentrations from the Munro NOEL dataset can be considered for thousands of data poor chemicals in absence of chemical-specific hazard data. The iTTCs can be compared against measured blood concentrations and against predicted whole-body or blood concentrations from multi-route and aggregate exposure scenarios for risk (or safety) based screening and priority setting assessment objectives. For example, while existing oral-dose based TTCs have been compared with high-throughput external exposure estimates for risk-based priority setting [20], the new iTTC can be used with internal aggregate exposure estimates using tools like the PROduction-To-EXposure High Throughput (PROTEX-HT) model [33]. Blackburn et al., [25] have previously proposed an in vitro derived iTTC of 1 µmol/L for cosmetics; however, this proposed value was associated with several caveats and exclusions making it difficult to directly compare with the new in vivo blood iTTC derived herein, i.e., 0.10 nmol/L-blood. We are not aware of any other reported iTTCs for blood and whole-body level.

Ignoring TK obscures the true potency and relative hazard of chemicals when they are expressed in terms of external doses, e.g., Fig. 3. While whole-body and blood concentrations are still only surrogates for concentrations at target sites corresponding to molecular initiating events and biological perturbations, the internal exposures are much more representative than the external administered doses. Possible exceptions are for chemicals that exert effects upon epithelial tissues at the site of contact or portal of entry for which the administered concentrations and applied doses may be better surrogates than systemic blood concentrations.

This study highlights opportunities for more broadly converting external exposure doses to internal exposure doses to better compare and rank chemicals for relative potency in combination with other lines of evidence to address uncertainty in hazard characterization. Internal concentrations corresponding to oral NOELs and the iTTC can be compared against other existing and emerging data sources; however, it is imperative that the values used in the comparisons are in equal units and dimensions, e.g., [4]. The new internal concentrations can be compared with in vitro bioactivity data, e.g., from ToxCast, provided in vitro toxicokinetics are also considered, e.g., [51] and with in vivo tissue and body residues corresponding to effect responses, e.g., critical body residues [52]. Future comparisons of internal doses corresponding with biological effect, perturbations and no effects obtained from in vivo, in vitro and in silico methods will help strengthen weight of evidence approaches when establishing chemical-specific hazard thresholds and provide opportunities for further advancing Adverse Outcome Pathways (AOPs) by quantitatively linking internal exposure to key events and key event relationships.

### Recommendations for future iTTCs

In future work, the new tiered TK methods can also be applied to other TTC datasets (e.g., acetylcholinesterase inhibitors, inhalation, and dermal data) to expand and better define the applicability domain of the new iTTCs. Multi-compartment PBTK models can also be considered when they can be parameterized with required TK data [29]. Future work to derive iTTCs should include a more intensive examination of in vitro, in vivo and in silico estimates for TK data used to parameterize the models and the development of validated QSARs for predicting in vitro or in vivo biotransformation rates and whole-body clearance in rodent species would be helpful. To address uncertainty in biotransformation rate data and to reduce unnecessary animal testing, long-term integrated testing strategies combining in vitro, in vivo and in silico methods for estimating biotransformation rates are strongly encouraged.

The emerging iTTC approach can also be aligned with general recommendations for improving the TTC approach, e.g., [53] and other on-going efforts to develop iTTCs [29]. There may be value in developing Cramer Class iTTCs in future work to better align with existing decision-trees, e.g., [14, 15]; however, we recommend a thorough evaluation of the in vivo NOEL data beforehand. Moreover, while the Cramer Class TTCs and other TTCs have proven useful, there are unique opportunities with iTTCs that could be explored for alternative classifications. Approaches to derive iTTCs for chemicals grouped by Mode of Action (MOA) or chemical class, may prove fruitful. The iTTC approach also fosters the capacity for improved inter-species comparisons and potential species sensitivities where uncertainties in inter-species toxicokinetics are greatly reduced. Finally, as discussed by Dankovic et al. [47] general approaches for applying uncertainty factors i.e., 100x, that seek to account for TK and toxicodynamic differences may or may not be sufficient to account for differences between animals and humans and thus examination of the application of uncertainty factors for extrapolating hazard data is warranted as more explicit TK modeling efforts are developed and applied.

## Supplementary Information

SUPPLEMENTARY INFORMATION

Supplementary Information

## Data Availability

The supplemental material includes additional details regarding the toxicity data, the models, and the chemical information as well as summary model output results used in this study.
