# Correction to: Developing an internal threshold of toxicological concern (iTTC)

**DOI:** 10.1038/s41370-023-00582-6

**Published:** 2023-07-13

**Authors:** Jon A. Arnot, Liisa Toose, James M. Armitage, Alessandro Sangion, Alexandra Looky, Trevor N. Brown, Li Li, Richard A. Becker

**Affiliations:** 1 ARC Arnot Research and Consulting Inc., Toronto, ON Canada; 2 Department of Physical and Environmental Sciences, University of Toronto Scarborough, Toronto, ON Canada; 3 Department of Pharmacology and Toxicology, University of Toronto, Toronto, ON Canada; 4 School of Public Health, University of Nevada Reno, Reno, NV USA; 5 American Chemistry Council, Washington, DC USA

Correction to: *Journal of Exposure Science & Environmental Epidemiology* 10.1038/s41370-022-00494-x, published online 08 November 2022

There were two typographical errors in units in the original publication. The corrections do not change the results of the research. The authors are grateful to Dr. Corie Ellison (Procter & Gamble) for bringing these errors to our attention.

The original article has been corrected.

In the original publication the units in the last sentence in the paragraph below were 22 nmol/L-blood (or 22 pmol/mL-blood), but they should have been 22 ng/L-blood (or 22 pg/mL-blood) as correctly written below:

Given the consensus in the iTTCs from the three models and the underlying uncertainty in the hazard and TK data a blood iTTC of 0.1 nmol/L (or pmol/mL-blood) is selected for organic chemicals that are not considered genotoxic and are not acetylcholinesterase inhibitors. This iTTC is therefore rather broadly applicable for a range of organic chemicals. Furthermore, because the new iTTC is expressed on a molar basis it can be readily converted to an iTTC expressed on a chemical mass basis by multiplying 0.1 nmol/L by the molar mass of the chemical of interest, for example when comparing to human exposure blood concentrations expressed in units of ng/L or pg/mL. As a first approximation, using the median value for molar mass in the dataset (220 g/mol) corresponds to a blood iTTC of 22 ng/L-blood (or 22 pg/mL-blood).

The axes in Figure 3 of the original publication should have been presented on a molar basis (µmol) rather than a mass basis (µg) because the data in the figure and the discussion of these data in the corresponding text are on a molar basis. The corrected axes for Figure 3 are:

**Fig. 3:**
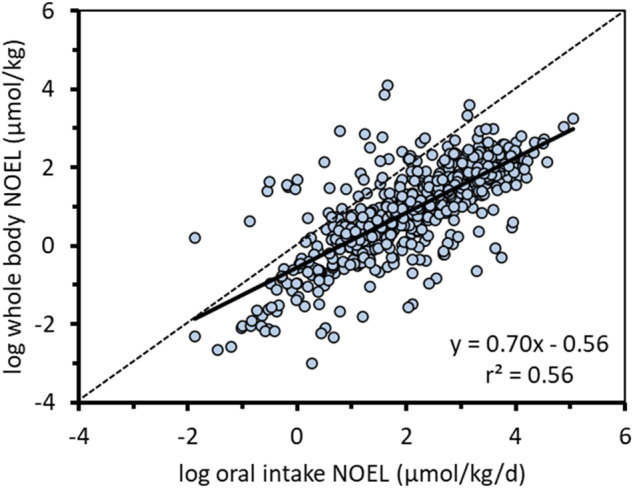
External and internal doses corresponding to NOELs in the Munro TTC database^11,39^. The dashed diagonal line represents the 1:1 line.

