# Supplementary material for: Developing an internal threshold of toxicological concern (iTTC)
Source: J Expo Sci Environ Epidemiol. 2022 Nov 8;32(6):877–84. doi: 10.1038/s41370-022-00494-x (PMC9731903; doi:10.1038/s41370-022-00494-x)
Supplement: Supplementary file 1 — SUPPLEMENTARY INFORMATION [file 41370_2022_494_MOESM1_ESM.pdf]

SUPPLEMENTARY INFORMATION: Developing an internal threshold of toxicological concern (iTTC)

Jon A. Arnot[1,2,3*], Liisa Toose[1], James M. Armitage[1], Alessandro Sangion[1,2], Alexandra Looky[1], Trevor N. Brown[1], Li Li[4], Rick Becker[5]

[1] ARC Arnot Research and Consulting Inc., Toronto ON

[2] Department of Physical and Environmental Sciences, University of Toronto Scarborough

[3] Department of Pharmacology and Toxicology, University of Toronto

[4] University of Nevada Reno, Reno NV

[5] American Chemistry Council, Washington DC

* Corresponding author: jon@arnotresearch.com

ORCID: 0000-0002-4295-4270

SECTIONS

S-1. Additional details on the Munro dataset
S-2. Details of the 1-CoPBTK model
S-3. In vitro-in vivo extrapolation (IVIVE) of biotransformation rate data
S-4. Summary of physical-chemical data used to parameterize the models
S-5. Evaluating the steady-state assumption in the Munro TTC database

S-1. Additional details on the Munro dataset

Some of the administered chemicals in the Munro database are salts (e.g., sodium cyclamate) and it is assumed that it is the organic moiety of these chemicals that exerts the toxic response (or lack thereof) in the original toxicity tests. Furthermore, doses administered as formulated salts will dissociate upon entering the gastrointestinal tract (GIT), such that the discrete organic moiety of the molecule being tested will have a different mass concentration than the administered mass. Based on reported CAS and SMILES information in the Bassan et al. [1] version of the Munro TTC database we obtained molar mass for the administered chemicals. All model calculations in this case study are for discrete organic molecules, i.e., the organic moiety. Therefore, we also obtained molar mass (and other chemical properties) based on the discrete organic moiety to convert the original NOELs based on chemical mass to molar concentrations for the discrete organic moiety, as necessary. Two chemicals (e.g., calcium formate, $Ca^{2+}[COO-]_2$), contain two equal organic moieties (e.g., formate, [COO-]) in each original test molecule. Therefore, in these cases the ensuing molar concentrations for the organic moiety were doubled to account for the quantity of presumed toxic agent. We identified that at least one of the test substances in the Munro database is Arochlor 1254, which is a mixture of various polychlorinated biphenyls (PCBs). The CAS that was used in the Bassan et al. [1] database is for a monochlorinated PCB; however, Arochlor 1254 is predominantly comprised of chlorinated biphenyls with an average chlorine content of 54% by mass instead of 19%. We selected a SMILES for a pentachlorobiphenyl (PCB 105) since this chemical structure more closely reflects the average properties of the Arochlor mixture than the monochlorobiphenyl.

S-2. Details of the 1-CoPBTK model

The general 1-CoPBTK modeling approach used here is generally consistent with other models used to estimate chemical uptake and elimination processes in mammals [2-6], except where noted. All tissues/organs are grouped into a single compartment. While there can be explicit consideration for absorption efficiencies or bioavailability at each portal of entry (e.g., lung, skin, gastro-intestinal tract) there is no explicit and initial consideration for chemical distribution in the organism; the chemical is instantaneously well-mixed throughout the entire body. The composition of the whole body is however still characterized in terms of key biological phases (e.g., adipose, phospholipids, proteins, water) that can be estimated from the volumes and compositions of individual tissues and is therefore broadly consistent with MCo-PBTK models [7]. The model is parameterized to representative physiological parameters for an adult laboratory rat as summarized in **Table S-1**.

Table S-1. Default parameter value for laboratory rat (1Co-TK model)

Parameter	Default Value	Notes/Comments
Size		
Mass	0.25 kg	
Proximate Composition		
Storage lipid	0.0800	At whole body level
Membrane lipid	0.0098	
Structural protein	0.1998	
Serum albumin	0.0024	
Water	0.7080	
Key Uptake Rates		
Inhalation	0.0075 m^3/h	
Ingestion (Food)	5.65e-7 m^3/h	5.4% of BW per day
Ingestion (Water)	1.18e-6 m^3/h	
Chemical Assimilation Efficiencies		
From lungs (E_A)	0.7	
From GIT (E_I)	*Chemical specific*	*f* of hydrophobicity (K_{OW}), see [8]
Proximate Composition of Diet		Commercial rat chow pellets (dry)
Storage lipid	0.04	
Membrane lipid	0.01	Overall composition of ingesta calculated as
Protein	0.24	a function of food and water ingestion rates
Carbohydrates	0.54	
Water	0.17	
Dietary Assimilation Efficiencies		
Storage lipid	0.95	
Membrane lipid	0.95	
Protein	0.75	
Carbohydrates	0.75	
Water	0.85	
Key Elimination Rates		
Exhalation	0.0075 m^3/h	
Fecal egestion	-	*f* of total ingestion & assimilation
Urinary elimination	-	*f* of total water ingestion & assimilation
Biotransformation	*Chemical specific*	
Growth rate constant	$6.25 \times 10^{-5}/h$	Equivalent to 0.0015 per d

The 1-CoPBTK model is coded in fugacity format [9, 10]; however, we present the equations in the more common rate constant format. The uptake and elimination of xenobiotics in a mammal can be expressed by the following mass balance equation:

$$dC_R/dt = k_{RI}C_{AG} + k_I C_I - (k_{RO} + k_E + k_R + k_B + k_{RL} + k_G + k_D)C_R \quad \text{(S-1)}$$

where dC_R/dt is the net change in concentration in the rat (µmol/kg) over time t (h), C_R is the concentration of the xenobiotic in the rat, k_{RI} is the respiration intake rate constant (L(kg·h)$^{-1}$), C_{AG} is the gaseous concentration (g·L^{-1}), k_I is the ingestion rate constant (kg(kg·h)$^{-1}$), and C_I is the concentration of the xenobiotic (µmol/kg) ingested. The rate constants (h^{-1}) corresponding to xenobiotic elimination via respiratory elimination, fecal egestion, renal excretion, biotransformation, and growth dilution are k_{RO}, k_E, k_R, k_B, k_G, respectively. Growth dilution is a "pseudo" elimination process in that the chemical is not actually eliminated from the organism, but the change in concentration of the chemical is a function of changes in biomass. The total first order xenobiotic elimination process is the sum of various individual elimination processes, i.e., $k_T = k_{RO} + k_E + k_R + k_B + k_G$.

At steady-state ($dC_R/dt = 0$), Equation S-1 is:

$$k_{RI}C_{AG} + k_D C_D = (k_{RO} + k_E + k_R + k_B + k_G)C_R \quad \text{(S-2)}$$

where the left side of equation quantifies chemical uptake into the rat through exposures to chemical in air, food and water and the right side of the equation quantifies parent chemical elimination from the rat.

Respiratory Uptake

The respiration intake rate constant k_{RI} is calculated as:

$$k_{RI} = E_A G_R / M_R \quad \text{(S-3)}$$

where E_A is the chemical transfer efficiency in the lung (unitless), G_R is the respiration rate (L/h), and M_R is the mass of the rat (kg). The estimated alveolar respiration rate is 70% of the total respiration rate and thus E_A is approximated as 0.7 [11, 12]. In the current model applications, there is no exposure to chemical in the air; however, this equation is required for calculating respiratory elimination, as per below.

Respiratory Elimination

The respiratory elimination rate constant k_{RO} (h^{-1}) is calculated as:

$$k_{RO} = k_{RI}/K_{HA} \quad \text{(S-4)}$$

where k_{RI} is the respiration intake rate constant (L-air/kg-rat/h; Eqn S-3) and K_{HA} is the rat-air partition coefficient (kg-rat/L-air) estimated as:

$$K_{HA} = (SL_R K_{OA}/\delta_L + PL_R K_{OA}/\delta_L + P_R \rho K_{OA} + SA_R K_{SaA}/\delta_{Sa} + W_R/K_{AW}) \quad \text{(S-5)}$$

SL_R is the storage (adipose) lipid mass fraction of the rat on a wet weight basis, PL_R is the phospholipid (membrane) mass fraction of the rat on a wet weight basis, P_R is the structural protein mass fraction of the rat on a wet weight basis, SA_R is the serum albumin mass fraction of the rat on a whole body wet weight basis, W_R is the water fraction of the rat, δ_L is the density of lipid and δ_{Sa} is the density of serum albumin. ρ is the proportionality constant expressing the storage capacity of protein to that of octanol [3, 13]. Following this approach for parameterization, K_{OA} and K_{AW} are the octanol-air and air-water partition

coefficients (dimensionless) for the chemical, respectively. The serum albumin-air partition coefficient (K_{SaA}) is calculated as the ratio of the serum albumin-water partition coefficient (K_{SaW}) divided by K_{AW}, where K_{SaW} is estimated from octanol-water partitioning using the relationships suggested by Endo and Goss [14].

For ionogenic organic chemicals (IOCs) that are ionized at pH 7.4 K_{OA} and K_{AW} in Equation S-5 are replaced by the chemical-specific distribution coefficients D_{OA} and D_{AW}, respectively and K_{SaA} is replaced by a distribution ratio (D_{SaA}). Distribution ratios for IOCs are determined using the Hendersen-Hasselbalch equation and scaling factors relating the partitioning of the charged form of the chemicals to those of the neutral form, e.g., [7].

Ingestion Uptake

The ingestion intake rate constant k_I (kg-ingested(kg-rat·h)$^{-1}$ is calculated as:

$$k_I = E_I G_I / M_R \quad \text{(S-6)}$$

where E_I is the chemical transfer efficiency from the gastrointestinal tract GIT (unitless), G_I is the ingestion rate (kg/h) and M_R is the rat mass (kg). The chemical uptake efficiency is based on a GIT residence time model developed by Arnot and Mackay [8] which has been parameterized here for mammals. **Table S-2** summarizes the E_I parameters for mammals. The 1Co-PBTK model includes a digestion model that simulates chemical biomagnification from the GIT into the body. This mechanistic biomagnification model is well-established in the ecological health literature [2, 3, 15-17] and explicitly considers the degree to which ingested materials are digested in the GIT and subsequently assimilated into the body, which along with the composition of the body, determines the fugacity gradient (i.e., the driving force for passive diffusion of chemical into the body).

Fecal Egestion

The fecal egestion rate constant k_E (h^{-1}) is:

$$k_E = G_F E_I K_{GH} / M_R \quad \text{(S-7)}$$

where G_F (kg-ww/h) is the fecal egestion rate, K_{GH} is the partition coefficient of the chemical between the GIT and the rat (kg-human/kg-feces), and E_I (unitless) is the chemical transfer efficiency between the GIT and the rat. G_F was calculated from the feeding rate G_I (kg-food/h), the digestibility of the diet, and the composition of the diet as:

$$G_F = ([(1-\varepsilon_L)L_I + (1-\varepsilon_P)P_I + (1-\varepsilon_C)C_I + (1-\varepsilon_W)W_I]G_I)/(L_G + P_G + C_G + W_G) \quad \text{(S-8)}$$

where ε_L, ε_P, ε_C and ε_W are the dietary absorption efficiencies of lipid, protein, carbohydrate, and water, respectively. L_G, P_G, C_G and W_G are the mass fractions (kg/kg) in the gut (feces) calculated below.,

The degree to which ingested nutrients are absorbed and assimilated by the body influences the degree to which chemicals are subsequently absorbed as reflected by K_{GH}. For neutral organics K_{GH} is calculated as:

$$K_{GH} = (L_G K_{OW}/\delta_L + P_G \rho K_{OW} + C_G \rho K_{OW} + W_G) / (L_R K_{OW}/\delta_L + P_R \rho K_{OW} + W_R) \quad \text{(S-9)}$$

where L_G, P_G, C_G, and W_G are the lipid, protein and carbohydrate, and water contents of the GIT, respectively, after digestion. For IOCs, K_{OW} is replaced D_{OW}. For simplicity in these equations fats and

phospholipids are lumped together as "total lipids" L. The sum of these fractions approach 1 and are dependent on the absorption efficiency for each component of the diet as:

$$L_G = [(1-\varepsilon_L)L_I] / [(1-\varepsilon_L)L_I + (1-\varepsilon_P)P_I + (1-\varepsilon_C)C_I + (1-\varepsilon_W)W_I] \quad \text{(S-10)}$$

$$P_G = [(1-\varepsilon_P)P_I] / [(1-\varepsilon_L)L_I + (1-\varepsilon_P)P_I + (1-\varepsilon_C)C_I + (1-\varepsilon_W)W_I] \quad \text{(S-11)}$$

$$C_G = [(1-\varepsilon_C)C_I] / [(1-\varepsilon_L)L_I + (1-\varepsilon_P)P_I + (1-\varepsilon_C)C_I + (1-\varepsilon_W)W_I] \quad \text{(S-12)}$$

$$W_G = [(1-\varepsilon_C)W_I] / [(1-\varepsilon_L)L_I + (1-\varepsilon_P)P_I + (1-\varepsilon_C)C_I + (1-\varepsilon_W)W_I] \quad \text{(S-13)}$$

Generic estimation of partitioning behaviour of IOCs is based on the behaviour of the neutral form and application of scaling factors for the charged form [13, 18].

Renal Clearance

We used two different sub-modules (models) for renal (urinary) elimination rate constant k_R (h^{-1}) in the current applications. The two basic approaches are equilibrium partitioning approach and the glomerular filtration rate approach as summarized elsewhere [7].

Growth dilution and dermal elimination

Growth dilution is considered a loss process, although parent chemical is not actually eliminated because of this process, rather the chemical concentration can become reduced in the increased mass and volume of the organism as it grows over time. A growth rate constant k_G (h^{-1}) of 6.25×10^{-5} [6] is included to account for some biological turnover and to include some dermal losses and this loss process is only relevant for very persistent chemicals.

Biotransformation

For simplicity and by necessity the biotransformation rates are assumed to follow first-order kinetics. First-order biotransformation rate constants (k_B, 1/d) result in a constant fraction of the mass of parent chemical being degraded per unit time. The in vivo whole body biotransformation half-lives ($HL_B = \ln 2/k_B$) were selected from in-house databases, e.g, [19] and literature searches for chemicals in the Munro TTC database for experimental mammals. In absence of empirical HL_B data, in vitro hepatocyte values and *in vitro-in vivo extrapolation* (IVIVE) models [20] and HL_B-QSARs [19, 21] were used to parameterize the 1Co-PBK models following the methods outlined in the main text. The 1-CoPBTK model was parameterized for a generic laboratory rat using HL_B input parameters scaled for body size (0.25 kg) using equation 4 in the main text.

Biotransformation half-life QSAR details including Applicability Domain methods

Two general types of methods were used in developing and validating the human biotransformation half-life QSARs (HL_B-QSARs [19, 21]) used in this study. One general method is the Iterative Fragment Selection (IFS) algorithm developed by Trevor Brown and colleagues which uses molecular fragments [19] and the second is based on holistic molecular descriptors selected by a Genetic Algorithm in the University of Insubria software QSARINS [21]. Both QSAR method predictions include Applicability Domain (AD) information. The AD of the IFS method is assessed determining the similarity of predicted chemical to those in the training dataset and how well the model fits those chemicals in the training dataset in terms of uncertainty levels (UL), i.e., UL ≥ 2 were considered "out" of the AD in the current work. The AD for the QSARINS models is estimated based on the range of the experimental response and of the

molecular descriptors of chemicals in the training sets. The QSARINS AD also accounts for the leverage giving a metric for chemical similarity with the training set and accounting for extrapolations.

Table S-2. The chemical dietary absorption efficiency model [8] and parameters for mammals [6] used in the 1-CoPBTK model.

**Parameters**	**Mammals**
$E_I = 1 - \exp\left(-\frac{\tau_G}{\tau_A}\right)$	
$\tau_G = \frac{1}{\left(\frac{1}{\tau_{rxn}} + \frac{1}{\tau_{trans}}\right)}$	
$\tau_A = VW + VOct * Kow * \left(\frac{1}{GOct * Kow} + \frac{1}{Gw}\right)$	
GOct	$3x10^{-5}$
GW	950
VOct	$6x10^{-6}$
VW	$1x10^{-5}$
Gut transport HL, $\boldsymbol{\tau_{trans}}$ (h)	8
Gut reaction HL, $\boldsymbol{\tau_{rxn}}$ (h)	$1x10^{12}$

S-3. In vitro-in vivo extrapolation (IVIVE) of biotransformation rate data

The general procedure applied for the extrapolation of in vitro biotransformation rate data to the whole body level (IVIVE) follows the published literature, e.g., [20] and a compositional approach for fraction unbound in assays and blood and volume of distribution. Most publications and databases report intrinsic in vitro clearances ($CL_{IN\ VITRO,\ INT}$). The subsequent steps are to i) convert intrinsic in vitro clearance ($CL_{IN\ VITRO,\ INT}$) to intrinsic in vivo clearance ($CL_{IN\ VIVO,\ INT}$), ii) convert intrinsic in vivo clearance ($CL_{IN\ VIVO,\ INT}$) to hepatic blood clearance (CL_H), and iii) convert hepatic blood clearance (CL_H) to whole-body biotransformation rate constant (k_B) as:

<u>Intrinsic in vitro clearance → Intrinsic in vivo clearance</u>

$$CL_{IN\ VIVO,INT} = CL_{IN\ VITRO,INT} \cdot HL \cdot LW \qquad \text{(S-14)}$$

where HL is the number of hepatocyte cells per g liver (10^6 cells / g liver) and LW is the liver weight as a fraction of the total body weight (g liver / g body weight).

<u>Intrinsic in vivo clearance → Hepatic blood clearance</u>

$$CL_H = \frac{Q_H \cdot f_U \cdot CL_{INVIVO,INT} \cdot \frac{f_{W,assay}}{f_{W,Bl}}}{Q_H + f_U \cdot CL_{INVIVO,INT} \cdot \frac{f_{W,assay}}{f_{W,Bl}}} \quad \text{(S-15)}$$

where Q_H is the amount of blood flowing to the liver (ml/h/g), f_U is the ratio of the unbound fractions in blood and the in vitro test system. Following Krause and Goss [20], the ratio of water contents in the assay and blood is also included as a term in the CL_H expression

Hepatic blood clearance → Whole body biotransformation rate constant

k_B is calculated from hepatic clearance (CL_H) by dividing by the estimated Volume of Distribution (referenced to blood) V_D (ml/g):

$$k_B = \frac{CL_H}{V_D}$$

$V_{D,Bl}$ is estimated as shown below

$$V_D = \frac{K_{BW}}{K_{BlW}}$$

where K_{BW} is the equilibrium partition coefficient between the organism and water which is estimated using partitioning data and whole-body composition, as shown below:

$$K_{BW} = f_{SL,B}K_{SW} + f_{PL,B}K_{MW} + f_{BP,B}K_{PW} + f_{SA,B}K_{BSA} + f_{W,B}$$

where $f_{SL,B}$ is the whole-body fraction of storage lipids, $f_{PL,B}$ is the whole-body fraction of phospholipids, $f_{BP,B}$ is the whole-body fraction of bulk (structural) protein, $f_{SA,B}$ is the whole-body fraction of serum albumin, $f_{W,B}$ is the whole-body fraction of water (see **Table S-1**) and K_{SW} is the storage lipid-water partition coefficient, K_{MW} is the membrane-water partition coefficient, K_{PW} is the bulk (structural) protein-water partition coefficient, and K_{BSA} is the bovine serum albumin-water partition coefficient. K_{BlW} is calculated the same way except using the proximate composition of blood (see **Table S-3**)

Unbound fractions in in vitro systems ($f_{U,assay}$) and blood ($f_{U,Bl}$)

Although various QSARs are available to estimate unbound fractions in in vitro systems (e.g., [22, 23]), we prefer the compositional approach described below because it is consistent with the approach applied to blood (i.e., a function of partitioning coefficients and proximate composition).

The compositional approach to estimate fractions unbound in in vitro assays is based on the composition of the assay (storage lipid, membrane lipid, protein, and water content) and partitioning data. The fraction unbound in the assay ($f_{U,\,assay}$) is estimated as shown below.

$$f_{U,assay} = \frac{f_W}{K_{assay-w}}$$

where f_W is the water content of the assay and $K_{assay\text{-}w}$ is the assay-water partition coefficient. The assay-water partition coefficient is calculated using the following expression:

$$K_{assay-w} = f_{SL}K_{SW} + f_{PL}K_{MW} + f_{BP}K_{PW} + f_W$$

where f_{SL},f_{PL} and f_{BP} are the storage lipid, phospholipid and protein content of the assay and K_{SW}, K_{MW} and K_{PW} are the storage lipid water, membrane water and protein water partition coefficients respectively.

The estimated composition of a HEP assay system is based on i) the volume of individual hepatocyte cells (3.4×10^{-12} L/cell), the composition of each cell and the number of cells/ml assay entered by the user. The default hepatocyte cell composition is 0.02, 0.045, 0.19 and 0.745 for storage lipids, phospholipids, protein, and water respectively. Assay compositions for HEP systems are summarized in **Table S-4**.

The unbound fraction in blood is calculated similarly, i.e., from water content and equilibrium partition coefficient between blood and water, K_{BlW}.

$$f_{U,BL} = \frac{f_{W,Bl}}{K_{BlW}}$$

<u>Summary Tables of Key Inputs for IVIVE for in vitro rat biotransformation data</u>

Table S-3. Summary of IVIVE parameters and default values for laboratory rats.

Parameter	Symbol	Value	Units
Liver weight (fraction of total BW)<br><br># of liver cells/g liver (HEP)	LW<br><br>HL	0.038<br><br>120	g liver/g BW<br><br>10^6 cells/g liver
Total cardiac output<br><br>Fraction of cardiac output to liver<br><br>Cardiac flow to liver	Q_C<br><br>LF<br><br>Q_H	Calc. [24]<br><br>0.183<br><br>Q_C x LF	L/h/kg (ml/h/g)<br><br>-<br><br>*Calc.*
Organism Composition<br>**See Table S-1**	-	-	-
Blood Composition<br>Storage lipid<br>Phospholipid<br>Bulk Protein<br>Serum albumin<br>Water	 $f_{SL,Bl}$<br>$f_{PL,Bl}$<br>$f_{SP,Bl}$<br>$f_{SA,Bl}$<br>$f_{W,Bl}$	 0.0013<br>0.0022<br>0.1600<br>0.0160<br>0.8205	

Table S-4. Assay composition for example hepatocyte (HEP) systems.

Assay System	Hepatocyte concentration (10^6 cell/ml)	Assay Phase	Fraction
HEP	1	Storage lipid<br>Phospholipid<br>Protein<br>Water	6.75e-05<br>1.52e-04<br>6.41e-04<br>9.99e-01

S-4. Summary of physical-chemical data used to parameterize the models

The generic 1-CoPBTK model can simulate TK for a monoprotic acid or base. Major acid and major base pKas were obtained from ACD Labs Classic pKa model predictions (Release 2019.2.1, Build 3285. 16 Jan 2020) and if multiple pkas (ionization centres) for a chemical exists then the strongest value at pH 7.4 was used (i.e., the chemical was treated as a monoprotic acid or base). Measured K_{OW} and K_{OA} values reported in the EPI Suite database [25] were selected preferentially over predicted chemical properties. In the absence of empirical data, predictions for K_{OW} and K_{OA} from EPI Suite [25], OPERA [26, 27] and pp-LFERS [28] and ACD Labs (Consensus LogP only) were averaged. Volume of distribution (V_D, L-blood/kg) was calculated using a compositional method. Chemical input parameters and summary output calculations are summarized in the accompanying XLS Supplemental Materials file.

S-5. Evaluating the steady-state assumption in the Munro TTC database

A comparison of the reported exposure durations and 4 x HL_T (i.e., time to achieve 95% of steady-state concentration) in hours is presented in **Figure S-1**.

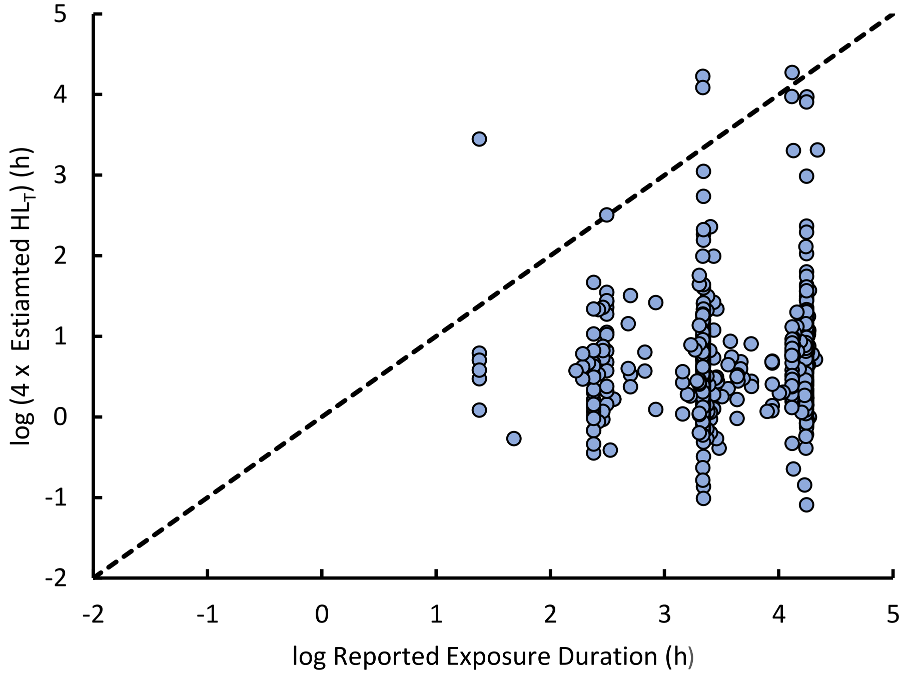

Figure S-1. Reported exposure durations (log units, h) vs 4 x Estimated HL_T (log units, h) for the Munro TTC database observations included in the analyses using the default model parameterization. The three chemicals for which 95% of steady-state may not have occurred are etretinate, octabromodiphenyl ether, pentabromodiphenyl ether.

References

1. Bassan A, Fioravanzo E, Pavan M, Stocchero M Applicability of physicochemical data, QSARs and read-across in Threshold of Toxicological Concern assessment. EFSA Supporting Publications 2011; 8: 159E.
2. Armitage JM, Gobas FAPC A terrestrial food-chain bioaccumulation model for POPs. Environmental Science & Technology 2007; 41: 4019 - 4025.
3. Kelly BC, Ikonomou MG, Blair JD, Morin AE, Gobas FA Food web-specific biomagnification of persistent organic pollutants. Science 2007; 317: 236-239.
4. Arnot JA, Mackay D Policies for chemical hazard and risk priority setting: can persistence, bioaccumulation, toxicity and quantity information be combined? Environmental Science and Technology 2008; 42: 4648-4654.
5. Arnot JA, Brown TN, Wania F, Breivik K, McLachlan MS Prioritizing chemicals and data requirements for screening-level exposure and risk assessment. Environmental Health Perspectives 2012; 120: 1565-1570.
6. Arnot JA, Toose L, Armitage JM, Embry M, Sangion A, Hughes L A weight of evidence approach for bioaccumulation assessment. Integrated Environmental Assessment and Management 2022; doi 10.1002/ieam.4583.
7. Armitage JM, Hughes L, Sangion A, Arnot JA Development and intercomparison of single and multicompartment physiologically-based toxicokinetic models: Implications for model selection and tiered modeling frameworks. Environment international 2021; 154: 106557.
8. Arnot JA, Mackay D The influence of chemical degradation during dietary exposures to fish on biomagnification factors and bioaccumulation factors. Environmental Science: Processes & Impacts 2018; 20: 86 - 97.
9. Paterson S, Mackay D A steady-state fugacity-based pharmacokinetic model with simultaneous multiple exposure routes. Environmental Toxicology and Chemistry 1987; 6: 395-408.
10. Cahill TM, Cousins I, Mackay D Development and application of a generalized physiologically based pharmacokinetic model for multiple environmental contaminants. Environ Toxicol Chem 2003; 22: 26-34.
11. U.S. EPA. *Exposure Factors Handbook.* U.S. Environmental Protection Agency: Washington, DC, 1997. Report no.: EPA/600/P-95/002B.
12. Hickie B, Mackay D, de Koning J Lifetime Pharmacokinetic Model for Hydrophobic Contaminants in Marine Mammals. Environmental Toxicology and Chemistry 1999; 18: 2622-2633.
13. Schmitt W General approach for the calculation of tissue to plasma partition coefficients. Toxicology in vitro : an international journal published in association with BIBRA 2008; 22: 457-467.
14. Endo S, Goss KU Serum albumin binding of structurally diverse neutral organic compounds: data and models. Chemical research in toxicology 2011; 24: 2293-2301.
15. Arnot JA, Gobas FAPC A food web bioaccumulation model for organic chemicals in aquatic ecosystems. Environmental Toxicology and Chemistry 2004; 23: 2343-2355.
16. Kelly BC, Gobas FAPC, McLachlan MS Intestinal Absorption and Biomagnification of Organic Contaminants in Fish, Wildlife, and Humans. Environmental Toxicology and Chemistry 2004; 23: 2324-2336.

17. Gobas FAPC, Wilcockson JB, Russell RW, Haffner GD Mechanism of biomagnification in fish under laboratory and field conditions. Environmental Science and Technology 1999; 33: 133-141.

18. Armitage JM, Erickson RJ, Luckenbach T, Ng CA, Prosser RS, Arnot JA *et al* Assessing the bioaccumulation potential of ionizable organic compounds: Current knowledge and research priorities. Environ Toxicol Chem 2017; 36: 882-897.

19. Arnot JA, Brown TN, Wania F Estimating screening-level organic chemical half-lives in humans. Environmental Science and Technology 2014; 48: 723-730.

20. Krause S, Goss KU In Vitro-in Vivo Extrapolation of Hepatic Metabolism for Different Scenarios - a Toolbox. Chemical research in toxicology 2018; 31: 1195-1202.

21. Papa E, Sangion A, Arnot JA, Gramatica P Development of human biotransformation QSARs and application for PBT assessment refinement. Food and Chemical Toxicology 2018; 112: 535-543.

22. Austin RP, Barton P, Cockroft SL, Wenlock MC, Riley RJ The influence of nonspecific microsomal binding on apparent intrinsic clearance, and its prediction from physicochemical properties. Drug metabolism and disposition: the biological fate of chemicals 2002; 30: 1497-1503.

23. Nichols JW, Huggett DB, Arnot JA, Fitzsimmons PN, Cowan-Ellsberry CE Toward improved models for predicting bioconcentration of well-metabolized compounds by rainbow trout using measured rates of in vitro intrinsic clearance. Environ Toxicol Chem 2013; 32: 1611-1622.

24. Dawson TH Allometric Relations and Scaling Laws for the Cardiovascular System of Mammals. Systems 2014; 2: 168-185.

25. U.S. EPA. Estimation Programs Interface (EPI) Suite for Microsoft® Windows, Ver. 4.11. In. Released November, 2017 ed. Washington, D.C.: U. S. Environmental Protection Agency, 2017.

26. Mansouri K, Grulke CM, Judson RS, Williams AJ OPERA models for predicting physicochemical properties and environmental fate endpoints. Journal of Cheminformatics 2018; 10: 10.

27. Williams AJ, Grulke CM, Edwards J, McEachran AD, Mansouri K, Baker NC *et al* The CompTox Chemistry Dashboard: a community data resource for environmental chemistry. Journal of Cheminformatics 2017; 9: 61.

28. Ulrich N, Endo S, Brown TN, Watanabe N, Bronner G, Abraham MH *et al*. UFZ-LSER database v 3.2.1 [Internet]. In. Leipzig, Deutschland, Helmholtz Zentrum für Umweltforschung - UFZ, 2017.
